# Interpretable machine learning to predict functional visual outcomes after the anti-VEGF loading phase for macular edema secondary to retinal vein occlusion: model development and temporal internal validation

**DOI:** 10.3389/fmed.2026.1837014

**Published:** 2026-05-18

**Authors:** Haiyue Yu, Juan Teng, Zhijian Yao, Qin Zhang, Tiangang Liu, Liming Tao

**Affiliations:** 1 Department of Ophthalmology, The Second Affiliated Hospital of Anhui Medical University, Hefei, Anhui, China; 2 Department of Ophthalmology, The Second People's Hospital of Bengbu, Bengbu, Anhui, China

**Keywords:** anti-VEGF therapy, machine learning, macular edema, retinal vein occlusion, SHAP, XGBoost

## Abstract

**Background:**

Functional visual outcomes in macular edema (ME) secondary to retinal vein occlusion (RVO) after the anti-vascular endothelial growth factor (VEGF) loading phase are highly variable. We aimed to develop and validate an interpretable prediction model integrating baseline clinical features and quantitative optical coherence tomography (OCT) biomarkers to identify RVO-ME patients at high risk of poor functional visual outcomes.

**Methods:**

This study retrospectively included 196 patients with RVO-ME. Adopting a rigorous temporal validation design, cases from 2021 to 2024 were assigned to the training set (*n* = 156), while those from 2025 served as an independent test set (*n* = 40). A least absolute shrinkage and selection operator (LASSO) regression was utilized to select key features from 15 candidate variables to facilitate the development and comparison of six ML models, such as XGBoost, random forest, and logistic regression. Finally, SHapley Additive exPlanations (SHAP) analysis was employed to provide a transparent interpretation of the decision-making logic for the optimal model. Poor functional visual outcomes were defined as post-loading BCVA < 0.5 decimal (>0.3 logMAR).

**Results:**

Initially, LASSO regression identified five key predictors: baseline BCVA, age, RVO subtype, subretinal fluid (SRF) cross-sectional area, and length of disorganization of retinal inner layers (DRIL). The XGBoost model was selected as the best-performing model, showing strong performance in the test set (AUC, 0.898; 95% CI, 0.797–1.000; sensitivity, 0.917; specificity, 0.812) at the default probability threshold of 0.5. SHAP analysis identified baseline BCVA as the primary prognostic driver and suggested a non-linear relationship with prognosis, with a critical risk threshold at approximately 1.14 logMAR (95% CI: 1.07–1.20). Additionally, a heterogeneous, non-linear interaction pattern was observed between advanced age and severe baseline visual impairment. SHAP analysis identified SRF cross-sectional area and DRIL length as independent contributors to model predictions after accounting for other included features.

**Conclusion:**

XGBoost showed encouraging performance for predicting poor functional visual outcomes in patients with RVO-ME after the anti-VEGF loading phase and maintained high sensitivity in the independent temporal test cohort. Furthermore, SHAP analysis suggested a non-linear relationship between baseline BCVA and prognosis, with a breakpoint at approximately 1.14 logMAR, and highlighted the independent predictive value of quantitative DRIL and SRF in determining functional visual recovery. Overall, this study provides a proof-of-concept for interpretable prediction in RVO-ME and may help identify patients at higher risk of poor short-term functional outcomes.

## 1 Introduction

Retinal vein occlusion (RVO) is a critical retinal vascular disorder worldwide, second only to diabetic retinopathy, and the primary driver of visual impairment is secondary macular edema (ME) (1). Currently, anti-vascular endothelial growth factor (VEGF) therapy has been established to be a standard of care for RVO-ME, which can achieve significant anatomical resolution (2). However, substantial heterogeneity in visual outcomes remains after anti-VEGF therapy in real-world clinical practice. Compared to relative gains in visual acuity (VA), the attainment of functional vision [best-corrected visual acuity (BCVA) ≥ 0.5] can reveal the vision-related quality of life in specific patients more accurately (3). Therefore, predicting functional visual outcome is clinically meaningful for assessing therapeutic benefit, guiding patient counseling, and informing subsequent management strategies.

Traditionally, standalone parameters such as baseline BCVA and central macular thickness (CMT) represent the predominant modalities for clinical prognostication of RVO-ME. However, various microstructural biomarkers on optical coherence tomography (OCT), such as DRIL, SRF, and external limiting membrane disruption, have demonstrated superior predictive value with advancements in imaging technologies (4, 5). Nevertheless, continuous quantification of key microstructural biomarkers has remained insufficiently emphasized in many previous studies, which may limit the capture of pathological heterogeneity inherent in quantitative features. Moreover, it should be noted that there exist potentially complex non-linear associations between these biomarkers and functional visual outcomes. Unfortunately, traditional linear frameworks, such as logistic regression, struggle to capture high-dimensional interactions and hence constrain their predictive performance (6, 7). Recently, pioneering studies have successfully advanced RVO-ME prognostication through machine learning (ML) (8–10); however, even in interpretable frameworks incorporating SHAP analysis (10), these models predominantly utilize categorical OCT features, which may limit the capture of pathological heterogeneity. Although ML has shown promise for prognostic modeling in complex ophthalmic diseases, the lack of routinely applicable interpretability tools in clinical deployment has limited its broader integration into routine practice (11). In addition, important challenges remain regarding validation strategy, selection of clinically meaningful endpoints, and more refined quantitative characterization of OCT biomarkers in routine clinical prediction.

Accordingly, to address the aforementioned clinical challenges, this study developed and validated an interpretable ML-based prognostic model by integrating baseline clinical characteristics with quantitative OCT biomarkers. In our study, multiple algorithms were leveraged to explore high-dimensional non-linear associations, coupled with the employment of a temporal internal validation design to assess model performance in an independent test set. The model was specifically designed to identify patients at baseline who were at high risk of failing to achieve functional vision (BCVA ≥ 0.5) after the anti-VEGF loading phase. Furthermore, the decision-making process was demystified by incorporating the SHapley Additive exPlanations (SHAP) method to intuitively quantify the prognostic contribution of each parameter. This visualizable tool can both assist clinicians in formulating personalized intervention strategies and provide an objective and transparent scientific foundation for medical decision-making and patient counseling.

## 2 Method

### 2.1 Study population

This single-center retrospective study was conducted based on the clinical records of patients with RVO-ME who were diagnosed and treated at The Second People’s Hospital of Bengbu between January 2021 and December 2025. Inclusion criteria: (1) treatment-naïve RVO-ME [including branch RVO (BRVO) and central RVO (CRVO); and hemi-RVO (HRVO) cases categorized as BRVO] with no prior anti-VEGF or corticosteroid therapy (4); and (2) patients receiving intravitreal conbercept or ranibizumab treatment with a completed three-dose loading phase. Exclusion criteria: (1) cases with concurrent ocular conditions affecting vision, such as diabetic retinopathy or age-related macular degeneration; (2) cases undergoing intraocular surgery within 3 months preceding anti-VEGF initiation; and (3) cases with poor OCT image quality (e.g., low signal strength or severe artifacts) precluding precise quantitative analysis. A temporal internal validation design was employed to evaluate model robustness and temporal generalizability. Specifically, patients from January 2021 to December 2024 formed the training set, while those from January 2025 to December 2025 served as an independent test set. To ensure statistical independence, only one eye per patient was included in the analysis; in cases of bilateral involvement, the eye at the initial presentation was selected. In accordance with the tenets of the Declaration of Helsinki, this study was approved by the Ethics Committee of The Second People’s Hospital of Bengbu (Approval No.: Ethical Review [2026] No. 3). The requirement for informed consent was waived by the Ethics Committee given the retrospective design and the de-identification of all patient data.

### 2.2 Data collection and outcome definition

In terms of clinical data collection and treatment protocols, this study systematically retrieved potential predictors of visual prognosis, including: (1) demographic and clinical characteristics (age, sex, disease duration, diagnosis subtype, drug type and history of hypertension and diabetes mellitus); and (2) baseline best-corrected visual acuity (BCVA) and OCT biomarkers. All patients underwent an initial loading phase consisting of three consecutive monthly intravitreal injections of either conbercept (0.5 mg/0.05 mL) or ranibizumab (0.5 mg/0.05 mL). BCVA was measured using the Standard Logarithmic VA Chart and recorded in decimal notation. Prior to statistical analysis, all these values were converted to the logarithm of the minimum angle of resolution (LogMAR) using the equation: LogMAR = −log10 (Decimal VA). For low-vision categories, counting fingers and hand motion were converted to 1.85 and 2.30 LogMAR, respectively, according to previously published estimates (12).

For OCT image acquisition and quantitative analysis, all OCT scans were obtained using the Heidelberg Spectralis SD-OCT system (Heidelberg Engineering, Heidelberg, Germany) following a standardized macular protocol (20° × 20° volume scan with 25 B-scans; ART = 9). The quantification of biomarkers was performed through horizontal transfoveal B-scan passing through the foveal center. Scans were excluded from quantitative analysis if the image quality score was <15 dB or if severe motion artifacts, blink artifacts, or media opacity precluded reliable retinal layer assessment. Image analysis was performed independently by two experienced retina specialists who were blinded to all clinical data. The specific parameters included vitreoretinal interface abnormalities, central macular thickness (CMT), the length of disorganization of the retinal inner layers (DRIL), the number of hyperreflective dots (HRD), the cross-sectional area of intraretinal fluid (IRF), the cross-sectional area of subretinal fluid (SRF), and the length of ellipsoid zone (EZ) disruption (13). Definitions and measurement methodologies are detailed in Supplementary Table S1 and Supplementary Figure S1.

Concerning reliability assessment, the inter-observer reliability was evaluated using Cohen’s Kappa and intra-class correlation coefficients for qualitative categories and quantitative metrics, respectively (Supplementary Table S2). A third senior consultant was invited to resolve discrepancies in qualitative assessments through independent adjudication. For quantitative metrics, the mean of the two initial measurements was used if the discrepancy was < 10%; otherwise, a third senior consultant was incorporated for adjudication to reach a final consensus.

Additionally, the primary outcome referred to the functional visual status at 4 weeks after the third injection (approximately 12 weeks from the first injection). Specifically, “poor functional visual outcomes” were defined as BCVA < 0.5 decimal (equivalent to > 0.3 LogMAR) after the loading phase.

### 2.3 Model construction and evaluation

#### 2.3.1 Data preprocessing and feature engineering

A complete-case analysis was performed without any data imputation. Categorical variables (e.g., sex and diagnosis subtype) were processed using one-hot encoding. To prevent data leakage, *Z*-score standardization parameters [mean and standard deviation (SD)] were calculated exclusively from the training set and subsequently applied to the test set. Following this, a least absolute shrinkage and selection operator (LASSO) regression analysis was employed on the training set only to identify the most predictive features and mitigate multicollinearity among the variables. In this process, five-fold cross-validation with a fixed random seed (seed = 122) was used to determine the penalty parameter (lambda), and the 1-standard error rule was applied to select the final lambda. Specifically, LASSO was fit on the full training set; the five selected features were fixed before the 5-fold CV hyperparameter search of each ML model. The test set (2025) was held out throughout. To assess the stability of LASSO-based feature selection, bootstrap resampling analysis with 100 iterations was performed on the training set, and feature selection frequencies were recorded. Furthermore, the number of final predictors was constrained with reference to the events-per-variable principle commonly used in regression-based prediction modeling (14). In the training set, 87 outcome events and 5 selected features yielded an EPV of 17.4. Given that this sample size remains relatively limited for more complex machine-learning models, strict regularization and depth-limiting constraints were applied during model tuning to reduce the risk of overfitting.

#### 2.3.2 Model construction and optimization

The selected features were incorporated to construct and comprehensively evaluate the predictive performance of six mainstream ML classifiers: traditional logistic regression (LR), support vector machine (SVM), multilayer perceptron (MLP), k-nearest neighbors (KNN), random forest (RF), and extreme gradient boosting (XGBoost). During training, the key hyperparameters of each algorithm (e.g., maximum tree depth, learning rate, number of neighbors, and regularization penalties) were optimized systematically using five-fold cross-validation coupled with a grid search strategy. Additionally, the optimization objective was set to maximize the area under the receiver operating characteristic (ROC) curve (AUC) to achieve optimal classification discrimination.

#### 2.3.3 Model performance evaluation and interpretability analysis

Model performance was comprehensively evaluated across three dimensions: (1) discrimination, assessed by generating ROC curves and calculating the AUC, F1-score, accuracy, sensitivity, and specificity. Classification metrics were calculated using the default probability threshold of 0.5, and no additional sampling strategies or class weighting were applied; (2) calibration, evaluated by plotting calibration curves and computing the Brier score, Hosmer–Lemeshow test, calibration slope, and calibration-in-the-large (CITL) to determine the concordance between predicted probabilities and observed outcomes; and (3) clinical utility, determined using decision curve analysis (DCA) to estimate the net clinical benefit across different threshold probabilities. To assess the sensitivity of the temporal split, an additional random-split sensitivity analysis based on 10 repeated random 80/20 splits of the full cohort was performed. Finally, the best-performing model was interpreted by the SHapley Additive exPlanations (SHAP) method, with the visualization of the feature importance, non-linear associations, and individualized prediction explanations.

### 2.4 Statistical analysis

All statistical analyses and modeling were performed using R 4.3.1. Following the evaluation of normality using the Shapiro–Wilk test, normally distributed continuous variables were expressed as mean ± SD and compared using the independent samples *t*-test. Meanwhile, non-normally distributed data were presented as median with interquartile range (IQR) and compared using the Mann–Whitney *U* test. Categorical variables presented in frequencies (percentages) were analyzed using the chi-square test. The machine learning pipeline was implemented utilizing several R packages, including *caret*, *glmnet*, *xgboost*, *randomForest*, *e1071*, *kknn*, *kernelshap, shapviz, and segmented*. Additionally, the ROC curve analysis was conducted using the *pROC* package. A two-sided *p*-value < 0.05 was considered statistically significant.

## 3 Results

### 3.1 Baseline characteristics

**Figure 1:**
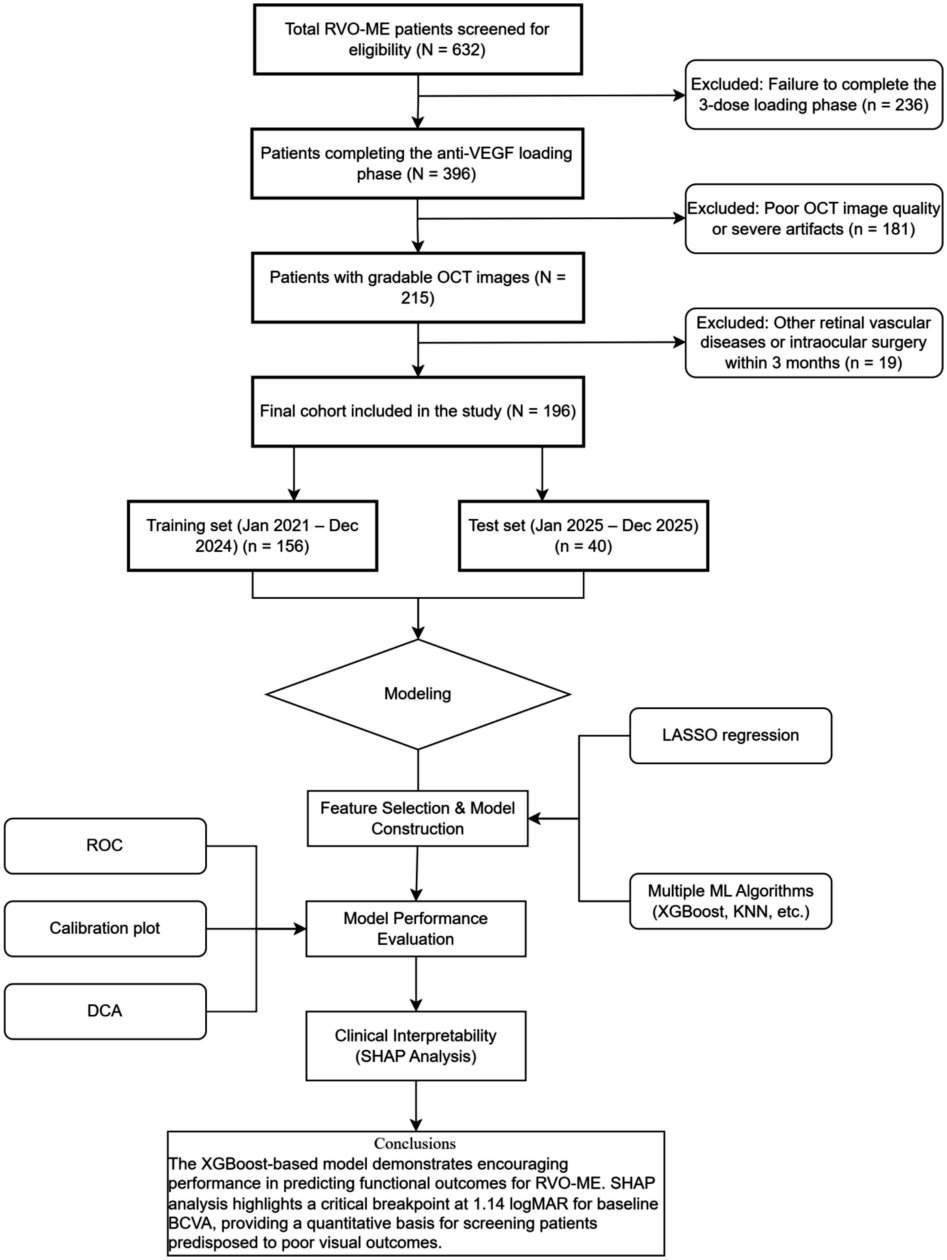
Flowchart of study population selection and the ML analysis pipeline.

Based on the strict inclusion and exclusion criteria, 632 patients with RVO-ME initially screened were sequentially excluded for the following reasons: failure to complete the three-dose anti-VEGF loading phase (*n* = 236); poor OCT image quality precluding precise quantitative analysis (*n* = 181); and concurrent ocular conditions affecting vision or a history of intraocular surgery within the preceding 3 months (*n* = 19). Ultimately, our final analysis incorporated 196 patients (196 eyes) with complete data. According to the temporal internal validation design, this study established two datasets: (1) the training set, including 156 patients treated between January 2021 and December 2024, and (2) the independent test set, including 40 cases treated between January and December 2025. The detailed screening flow is illustrated in Figure 1. Baseline characteristics of patients excluded due to incomplete loading phase completion and poor OCT quality are presented in Supplementary Tables S3, S4, respectively. The incomplete-loading group showed no significant differences in the available baseline characteristics. In contrast, the poor-OCT-quality group had worse baseline BCVA and a higher proportion of CRVO than the included cohort, suggesting potential selection bias related to OCT quality exclusion.

The mean age of the overall cohort was 59.56 ± 11.53 years, with males comprising 49.0%. BRVO and CRVO accounted for 61.2 and 38.8% of the enrolled cases, respectively. Meanwhile, 47.4% of the patients were treated with conbercept and 52.6% with ranibizumab. No statistically significant differences were observed in baseline characteristics, except for HRD (*p* = 0.007), between the training and test sets. To further assess potential distributional drift, a Kolmogorov–Smirnov test was performed for HRD, confirming a significant difference between the training and test sets (*D* = 0.272, *p* = 0.012). In addition, diabetes showed a borderline imbalance between the two cohorts. These findings suggested that the two cohorts were generally comparable in demographic and clinical characteristics, despite the difference in HRD and the borderline imbalance in diabetes. Baseline demographic and clinical characteristics of both groups are detailed in Table 1.

**Table 1:** Baseline characteristics in the training and test sets.

Characteristic	**Level**	**Overall**	**Train**	**Test**	***p***
*n*		196	156	40	
Age, years [mean (SD)]		59.56 (11.53)	59.85 (11.42)	58.42 (12.06)	0.488
Sex (%)	Male	96 (49.0)	76 (48.7)	20 (50.0)	1
	Female	100 (51.0)	80 (51.3)	20 (50.0)	
Diagnosis (%)	CRVO	76 (38.8)	62 (39.7)	14 (35.0)	0.713
	BRVO	120 (61.2)	94 (60.3)	26 (65.0)	
Course, days (median [IQR])		30.00 [10.00, 60.00]	30.00 [14.00, 60.00]	25.00 [9.25, 90.00]	0.629
Drug (%)	Conbercept	93 (47.4)	70 (44.9)	23 (57.5)	0.211
	Ranibizumab	103 (52.6)	86 (55.1)	17 (42.5)	
Diabetes (%)	No	171 (87.2)	140 (89.7)	31 (77.5)	0.071
	Yes	25 (12.8)	16 (10.3)	9 (22.5)	
Hypertension (%)	No	44 (22.4)	35 (22.4)	9 (22.5)	1
	Yes	152 (77.6)	121 (77.6)	31 (77.5)	
BCVA (median [IQR])		0.92 [0.52, 1.30]	0.92 [0.52, 1.30]	0.96 [0.70, 1.30]	0.649
Vitreoretinal Abnormalities (%)	No	173 (88.3)	137 (87.8)	36 (90.0)	0.915
	Yes	23 (11.7)	19 (12.2)	4 (10.0)	
CMT, μm (median [IQR])		564.50 [383.75, 830.00]	561.50 [387.00, 816.50]	591.50 [352.00, 886.00]	0.953
DRIL, μm (median [IQR])		4242.50 [2362.75, 5839.50]	4095.50 [2233.25, 5811.25]	4802.00 [3126.75, 5849.25]	0.284
HRD (median [IQR])		35.50 [16.75, 70.50]	42.50 [18.75, 77.00]	25.00 [10.00, 52.00]	0.007 ^*^
SRF, mm^2^ (median [IQR])		0.00 [0.00, 0.25]	0.00 [0.00, 0.25]	0.00 [0.00, 0.22]	0.342
IRF, mm^2^ (median [IQR])		0.10 [0.03, 0.22]	0.11 [0.04, 0.23]	0.08 [0.00, 0.16]	0.093
EZ Disruption Length, μm (median [IQR])		1722.00 [539.25, 3656.50]	2081.00 [553.25, 3816.50]	1356.00 [358.50, 2661.00]	0.129
Visual outcome (%)	Good	85 (43.4)	69 (44.2)	16 (40.0)	0.762
	Poor	111 (56.6)	87 (55.8)	24 (60.0)	

Values are presented as *n* (%) for categorical variables, mean (standard deviation, SD) for normally distributed continuous variables, and median [interquartile range, IQR] for non-normally distributed continuous variables. *p < 0.05.

### 3.2 Feature selection

**Figure 2:**
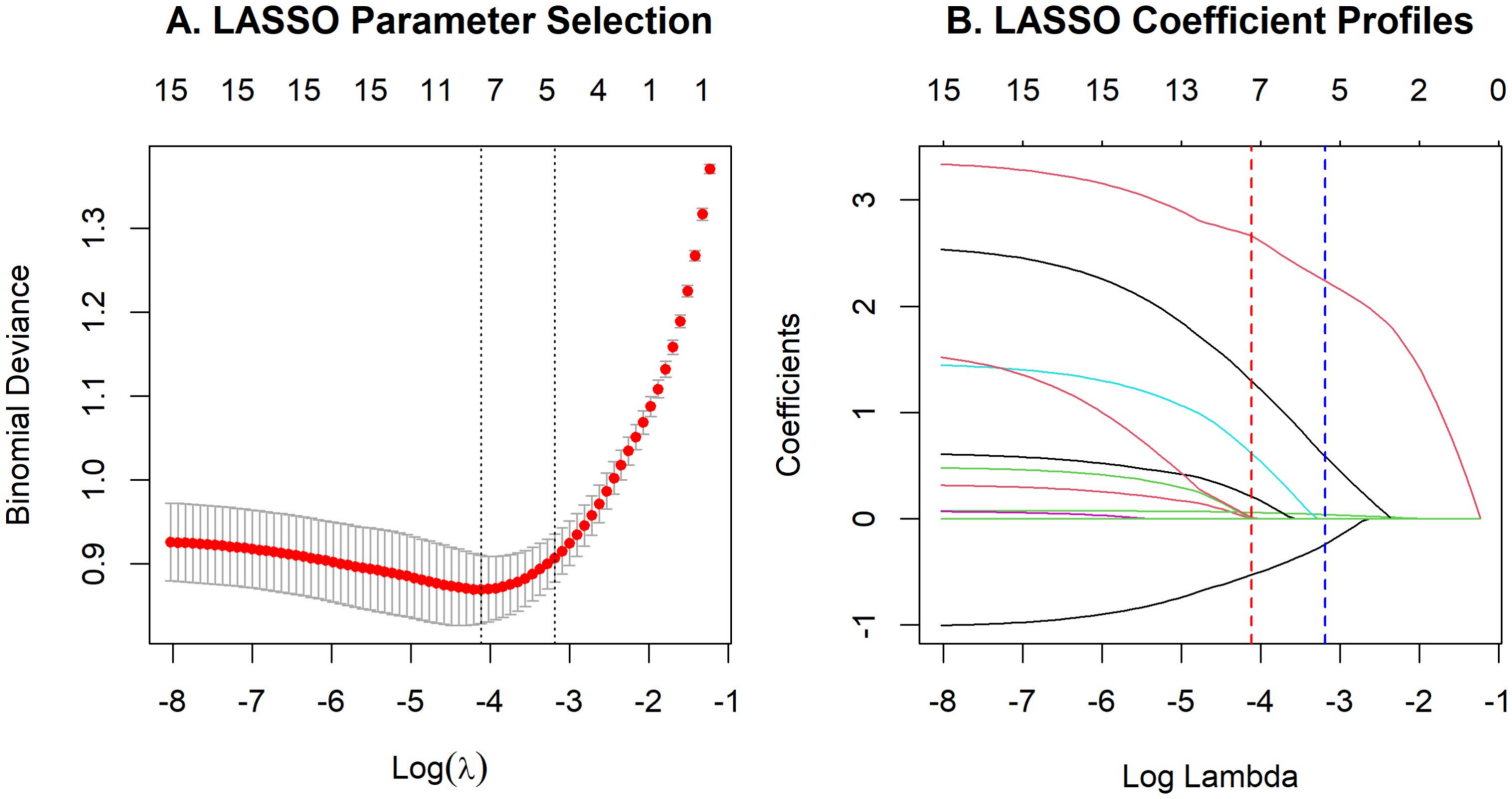
Feature selection using the LASSO binary logistic regression model. **(A)** LASSO parameter selection. The binomial deviance curve was plotted against the log(*λ*) sequence, and the tuning parameter λ was selected using 5-fold cross-validation. The left and right vertical dotted lines indicate λ_min_ and λ_1se_, respectively. **(B)** LASSO coefficient profiles. Each curve represents the coefficient of one of the 15 candidate variables varying with log(λ). The red and blue dotted lines indicate λ_min_ and λ_1se_, respectively. The λ_1se_ solution was selected for feature reduction and retained 5 non-zero coefficients.

A LASSO logistic regression model was employed to identify the features most predictive of functional visual outcomes in RVO-ME. With the use of a penalty function shrinking the coefficients of irrelevant variables to precisely zero, this method achieves dimensionality reduction and feature selection. A 5-fold cross-validation with a fixed random seed (seed = 122) was utilized to determine the penalty parameter (*λ*). According to the 1-standard error rule, the final λ value was 0.041 (Figure 2). Consequently, five baseline features (i.e., age, diagnosis subtype, BCVA, DRIL, and SRF) with non-zero coefficients were retained for subsequent modeling. Bootstrap resampling analysis (100 iterations) further supported the stability of feature selection, with selection frequencies of 100% for BCVA, 100% for SRF, 99% for age, 91% for diagnosis subtype, and 69% for DRIL. In contrast, HRD was selected in 52% of bootstrap samples, indicating lower selection stability and further supporting its exclusion from the final feature set.

### 3.3 Model construction and validation

Six ML classifiers, i.e., LR, SVM, MLP, KNN, RF, and XGBoost, were constructed and evaluated based on the selected features as described above. In addition, a baseline BCVA-only model was included as a benchmark comparator. During training, the key hyperparameters were systematically optimized using the five-fold cross-validation coupled with a grid search strategy, with optimal configurations detailed in Supplementary Table S5. The detailed performance metrics of each model are summarized in Table 2.

**Table 2:** Discrimination, classification, and calibration performance of the baseline and candidate models in the training and test sets.

Model name	AUC (95% CI)	Accuracy	Sensitivity	Specificity	Brier Score	HL Test (p)	Slope	CITL
Baseline (BCVA-only) (train)	0.853(0.790–0.916)	0.814	0.885	0.725	0.150	0.001	1	0
Baseline (BCVA-only) (test)	0.798(0.642–0.954)	0.800	0.875	0.688	0.175	0.014	0.708	0.069
LR (train)	0.897(0.848–0.946)	0.827	0.839	0.812	0.128	0.438	1	0
LR (test)	0.875(0.770–0.980)	0.775	0.833	0.688	0.144	0.890	1.119	−0.031
SVM (train)	0.898(0.850–0.946)	0.814	0.805	0.826	0.131	0.197	1.214	0.203
SVM (test)	0.865(0.756–0.973)	0.725	0.75	0.688	0.147	0.934	1.470	0.190
MLP (train)	0.932(0.894–0.969)	0.840	0.839	0.841	0.104	0.422	1.212	0.003
MLP (test)	0.870(0.759–0.980)	0.775	0.792	0.750	0.140	0.307	1.109	0.283
KNN (train)	0.882(0.830–0.935)	0.801	0.828	0.768	0.141	0.741	1.418	0.066
KNN (test)	0.901(0.809–0.993)	0.800	0.833	0.750	0.147	0.503	2.692	0.155
RF (train)	0.995(0.989–1.000)	0.962	0.966	0.957	0.044	0.041	3.686	0.088
RF (test)	0.872(0.753–0.992)	0.825	0.833	0.812	0.144	0.368	1.261	0.391
XGBoost (train)	0.947(0.916–0.978)	0.872	0.897	0.841	0.096	0.397	1.546	−0.075
XGBoost (test)	0.898(0.797–1.000)	0.875	0.917	0.812	0.128	0.790	1.658	−0.052

Performance metrics were calculated using the default probability threshold of 0.5. A non-significant Hosmer–Lemeshow test indicates no evidence of lack of fit. AUC, area under the receiver operating characteristic curve; CI, confidence interval; HL, Hosmer–Lemeshow; CITL, calibration-in-the-large; LR, logistic regression; SVM, support vector machine; MLP, multilayer perceptron; KNN, k-nearest neighbors; RF, random forest; and XGBoost, extreme gradient boosting.

**Figure 3:**
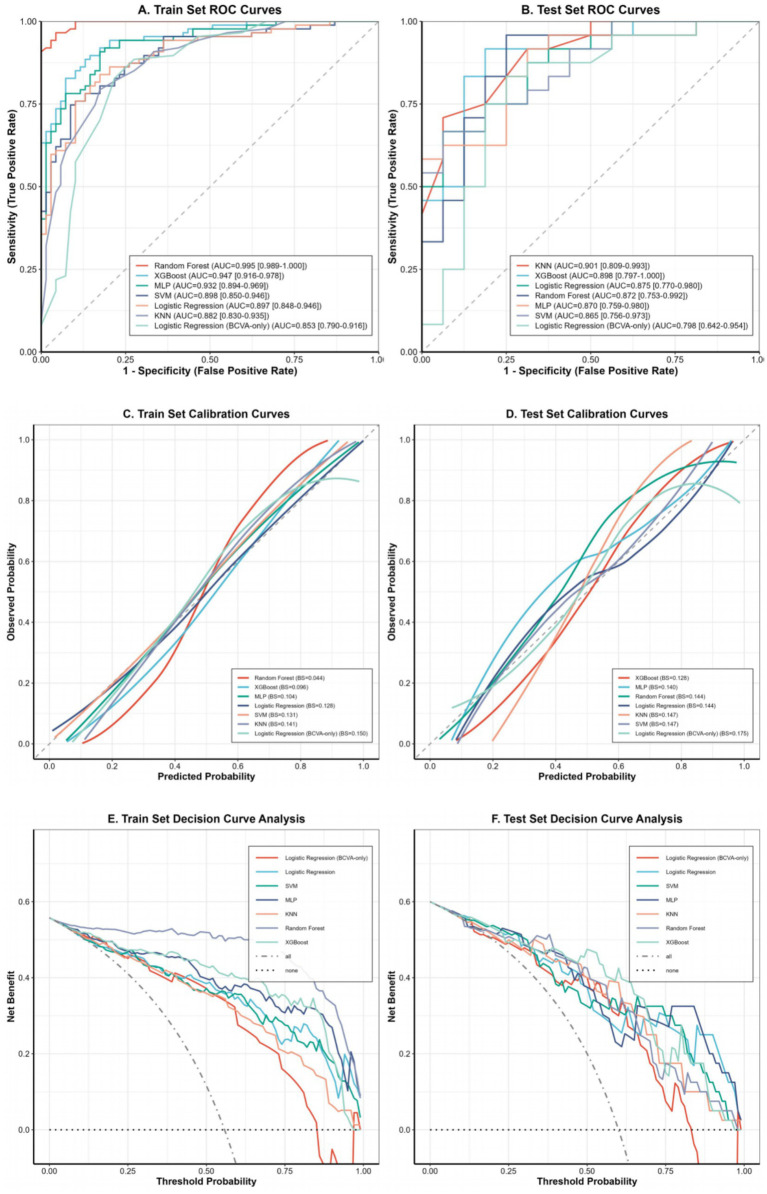
Comprehensive performance evaluation of the baseline and candidate predictive models. **(A,B)** ROC curves of the models in the training and test sets, respectively. **(C,D)** Calibration curves demonstrating the agreement between predicted probabilities and actual observations in the training and test sets (BS: Brier score). **(E,F)** DCA showing the clinical net benefit of the models across different threshold probabilities in the training and test sets.

Among the six candidate models, all achieved training-set AUCs above 0.88 (Figures 3A,B). In the independent test set, KNN and XGBoost showed the highest discrimination, with AUCs of 0.901 (95% CI: 0.809–0.993) and 0.898 (95% CI: 0.797–1.000), respectively, whereas the BCVA-only baseline model yielded a lower AUC of 0.798. Although KNN had a numerically slightly higher AUC, the difference between KNN and XGBoost was not statistically significant (DeLong *p* = 0.958). At a matched specificity of 0.80, XGBoost showed higher sensitivity than KNN (0.917 vs. 0.767). Moreover, XGBoost showed a more favorable calibration profile (Figures 3C,D and Table 2), with a calibration slope of 1.658 and a CITL of −0.052, suggesting limited overall miscalibration although some deviation from the diagonal remained at higher predicted probabilities. Therefore, XGBoost was selected as the best-performing model for subsequent interpretability analysis. Bootstrap-based stability estimation using 1,000 resamples of the independent test set yielded an AUC of 0.896 (95% CI: 0.767–0.992) (Supplementary Table S6). In addition, random-split sensitivity analysis based on 10 repeated 80/20 splits showed similar performance, with a mean AUC of 0.899 ± 0.047, sensitivity of 0.877 ± 0.071, and specificity of 0.806 ± 0.068 (Supplementary Table S6). In decision curve analysis (Figures 3E,F), at clinically relevant threshold probabilities of 20, 40, and 60%, XGBoost showed net benefits of 0.531, 0.475, and 0.425, respectively. Compared with the “intervene-all” strategy, the corresponding net benefit gains were 0.031, 0.142, and 0.425, respectively. Overall, XGBoost showed competitive net benefit at 20% and the highest net benefit at 40 and 60%.

### 3.4 SHAP analysis and model interpretation

**Figure 4:**
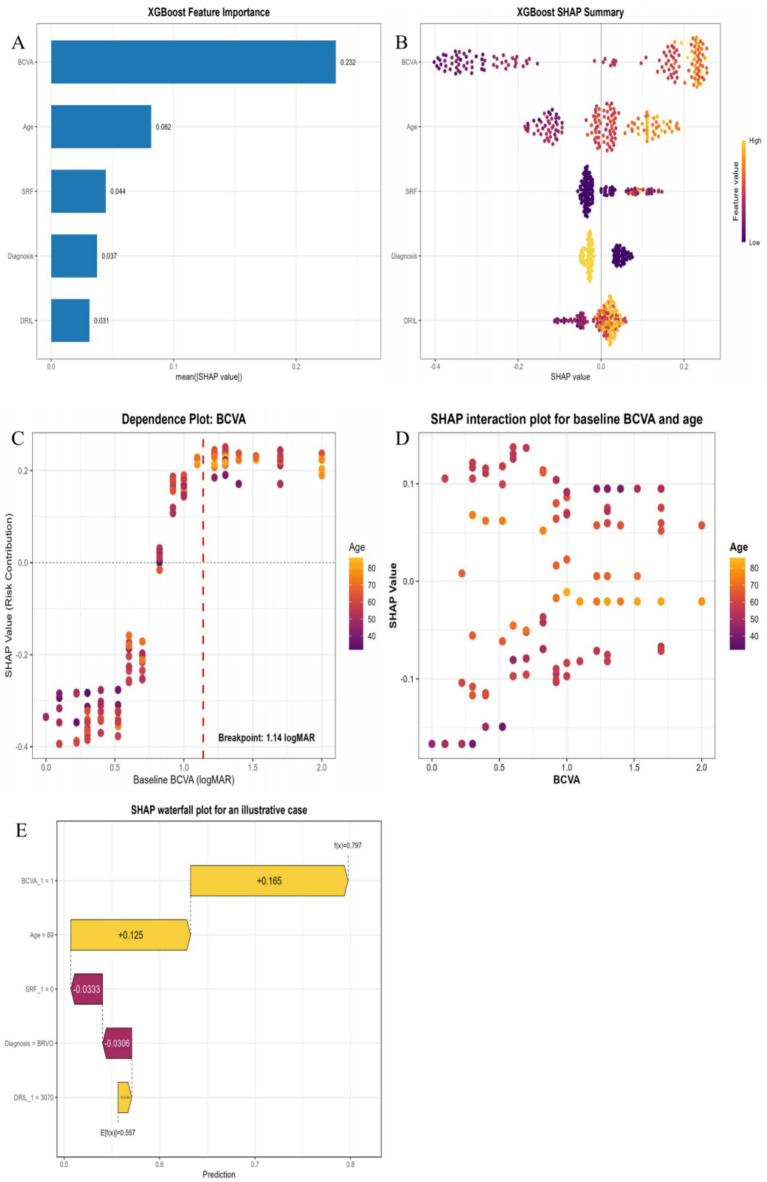
Interpretability analysis of the XGBoost model using the SHAP method. **(A)** Global feature importance ranking based on the mean absolute SHAP values. **(B)** SHAP summary plot illustrating the directional impact of features. Each dot represents a patient; while yellow and purple colors indicate higher and lower feature values, respectively. **(C)** SHAP dependence plot for baseline BCVA, color-coded by age. Segmented regression identified a breakpoint at 1.14 logMAR (95% CI: 1.07–1.20). **(D)** SHAP interaction plot for baseline BCVA and age. The interaction analysis suggested a heterogeneous, non-linear interaction pattern. **(E)** SHAP waterfall plot for an illustrative patient (Patient #6), aiming at visualizing the contribution of each feature to the final individualized prediction.

Finally, the XGBoost model was interpreted using the SHAP method. Global feature importance (Figure 4A) identified baseline BCVA as the most influential predictor, followed by age, SRF, diagnosis subtype, and DRIL. The SHAP summary (swarm) plot (Figure 4B) further corroborated the directional effects of these variables, with poor baseline BCVA (higher logMAR values) and advanced age associated with an increased risk of poor visual outcomes. Notably, the SHAP dependence plot for baseline BCVA (Figure 4C) showed a non-linear relationship with model output, while additional SHAP dependence plots for the other final predictors are provided in Supplementary Figure S2. Segmented regression identified a breakpoint at 1.14 logMAR (95% CI: 1.07–1.20). The SHAP interaction plot (Figure 4D) suggested a heterogeneous, non-linear interaction pattern between age and baseline BCVA, with local positive interaction values more frequently observed among older patients with worse baseline BCVA. In addition, a SHAP waterfall plot (Figure 4E) was generated to visualize the contributions of specific risk and protective factors to the predicted outcome for an illustrative patient.

## 4 Discussion

In this study, based on the XGBoost algorithm, we developed an interpretable clinical prediction model integrating baseline clinical characteristics with quantitative OCT biomarkers and subjected it to temporal internal validation. The model showed encouraging performance for predicting poor functional visual outcomes (decimal BCVA < 0.5) in RVO-ME patients after the anti-VEGF loading phase. First, XGBoost showed favorable overall performance in the independent temporal test set. It achieved high discrimination (AUC = 0.898) together with high sensitivity (0.917), and was selected as the best-performing model based on its overall discrimination, calibration, and decision-curve performance rather than AUC alone. Second, SHAP analysis suggested a non-linear threshold pattern for baseline BCVA and highlighted DRIL and SRF as important contributors to model predictions after accounting for the other included features.

Although the BCVA-only benchmark showed reasonable performance, the full model numerically outperformed it overall and demonstrated favorable net benefit within clinically relevant threshold ranges. These findings suggest that quantitative OCT biomarkers may provide incremental prognostic information beyond baseline visual acuity alone, although this incremental gain should still be interpreted cautiously given the limited size of the temporal test cohort.

Importantly, given the ensemble learning architecture of XGBoost, it achieved a superior balance between sensitivity and accuracy, although both this classifier and KNN yielded comparable AUCs. Compared with the distance metric-dependent KNN, XGBoost may be more robust to noise and better suited to heterogeneous clinical data (15, 16). In terms of model generalization, XGBoost showed a smaller train-to-test performance gap than Random Forest (AUC: 0.946 to 0.898 vs. 0.995 to 0.872), suggesting better generalization consistency. In small cohorts, Random Forest may be more prone to fitting noise through deeper or less constrained trees, whereas XGBoost incorporates regularization and explicit depth-limiting constraints (Supplementary Table S5), which may help control model complexity and reduce overfitting. Furthermore, traditional linear models that have been commonly applied in previous studies (17, 18) primarily assume linear relationships between clinical features and visual outcomes, which may not adequately capture the non-linear patterns observed in this study. By leveraging the architectural advantages of tree-based models (19), XGBoost can effectively overcome this limitation through the capture of complex clinical threshold effects and precise identification of non-linear feature interactions that elude traditional models. Additionally, the incorporation of SHAP-based interpretation tools enabled visualization of feature importance, non-linear associations, and individualized prediction explanations, thereby improving model interpretability in a clinically meaningful way (20).

Such robust predictive performance is largely attributable to the precise extraction of core prognostic features by the model. The SHAP analysis further clarified the key contributors to model predictions, and the observed patterns were broadly consistent with current ophthalmic pathophysiological understanding. First, baseline BCVA was identified as the most influential predictor of poor functional visual outcomes in the model (18). Generally, baseline BCVA can essentially embody the cumulative extent of retinal structural and functional impairment. Notably, SHAP analysis suggested a non-linear relationship between baseline BCVA and model output, and segmented regression identified a breakpoint at 1.14 logMAR (95% CI: 1.07–1.20). In addition, the SHAP interaction plot suggested a heterogeneous, non-linear interaction pattern between age and baseline BCVA. While SHAP identifies statistical contributors rather than causal mechanisms, the observed age–BCVA interaction pattern is consistent with known age-related retinal changes. This pattern reflects the threshold limits of retinal functional recovery, indicating the onset of irreversible structural and functional remodeling. Furthermore, advanced age further amplified the risk of poor functional visual outcomes at equivalent levels of baseline visual impairment. Its underlying mechanisms can be interpreted from two aspects as follows. First, age-related retinal arteriosclerosis may reduce vascular compliance, thereby exacerbating retinal ischemia and hypoxia (21); second, the age-dependent decline in retinal pigment epithelium pump function and metabolic reserve may compromise the efficiency of fluid resolution and ischemic repair (22). Consequently, patients with a baseline BCVA worse than 1.14 logMAR, particularly those of advanced age, may warrant consideration in prospective trials evaluating risk-stratified intensification protocols. Finally, the model assigned a higher predicted risk to CRVO than to BRVO, which is consistent with the more extensive retinal ischemia, hypoxia, and blood-retinal barrier disruption typically observed in CRVO (23).

However, it may be insufficient to fully elucidate the prognostic heterogeneity of RVO when relying solely on macroscopic clinical features. In our study, after accounting for the other included features (e.g., age, diagnosis subtype, and baseline BCVA), DRIL and SRF remained important contributors to model predictions, reflecting additional pathophysiological information underlying visual impairment. Specifically, DRIL—defined as the loss of identifiable boundaries among any two of the ganglion cell-inner plexiform layer complex, inner nuclear layer, and outer plexiform layer on OCT images—essentially mirrors an ischemic state within the inner retina (24, 25). Consistent with previous studies (26, 27), increased DRIL exhibited a strong positive correlation with poor functional visual outcomes, as indicated by the SHAP analysis. Furthermore, SRF serves as another critical predictor of poor outcomes. Because baseline SRF cross-sectional area was highly skewed, with many eyes showing no detectable SRF, its predictive contribution in the model should be interpreted as reflecting both the distinction between absent and present SRF and, among SRF-positive eyes, the extent of fluid accumulation. Additional SHAP dependence plots for the final predictors, including SRF, are provided in Supplementary Figure S2. A substantial volume of SRF is not merely a pathological hallmark of severe microvascular leakage and retinal pigment epithelium pump decompensation (28); the ensuing persistent subretinal detachment can also trigger irreversible apoptosis of photoreceptors, thereby hindering visual recovery (29). Consequently, by quantifying these OCT biomarkers, our model provides a more objective morphological basis for clinical risk stratification.

Recently, Liang C et al. (10) also developed and validated a similar model for clinical prediction, and their findings were broadly consistent with ours. However, as summarized in Table 3, the present study still provides complementary value in several important aspects. First, this study employed a temporal internal validation design, in contrast to the conventional random split-sample approach adopted by most studies. Its resilience against potential data drift was evaluated through the validation of the model on an independent chronological cohort (the year 2025). Although the 2025 test cohort was relatively small, the additional random-split sensitivity analysis showed similar performance, providing supportive evidence for the overall stability of our findings. Second, this study characterized key OCT microstructural biomarkers continuously and quantitatively, moving beyond the traditional framework of simple qualitative categorization. Building upon a maximal preservation of the heterogeneity of pathological information, this strategy captured and confirmed the independent predictive value of SRF for poor prognosis—a crucial detail frequently obscured in routine qualitative assessments. Notably, while our model demonstrated favorable sensitivity and specificity, we acknowledge that these performance gains may be linked to cohort variations and the specific features selected. Therefore, rather than replacing prior work, our study provides complementary evidence under temporal internal validation and a quantitatively characterized OCT framework.

**Table 3:** Comparison between Liang et al. (10) and the current study.

Comparison item	Liang et al. (10)	Current study
Population (n)	259	196
Loading-phase completion	Not required	Mandatory (3-dose completion)
Outcome type	Binary	Binary
Functional threshold	BCVA ≥ 0.5 decimal	BCVA ≥ 0.5 decimal
Validation strategy	Random split (7:3)	Temporal internal validation
Core predictors	Age, subtype, BCVA, DRIL, SBP	Age, subtype, BCVA, DRIL, SRF
OCT biomarker format	Predominantly categorical (except CMT)	Quantitative continuous measures
AUC	0.91	0.90
Sensitivity	0.88	0.92
Specificity	0.73	0.81

Performance metrics are reported as presented in the respective studies and should be interpreted in the context of differences in cohort composition, treatment standardization, and validation strategy.

Despite these encouraging findings, several limitations should be acknowledged. First, the retrospective design may have introduced selection bias, as patients excluded for poor OCT quality or incomplete loading-phase completion may represent a more severe or less treatment-compliant subgroup; the use of decimal visual acuity charts rather than ETDRS letter scores may also limit comparability with international studies. Second, although temporal internal validation was adopted, the 2025 test cohort was small (*n* = 40), leading to imprecise performance estimates, as reflected by the wide confidence intervals; in addition, the borderline imbalance in diabetes prevalence between the temporal cohorts may reflect clinically relevant cohort heterogeneity. Thus, the additional random-split sensitivity analysis should be regarded as supportive rather than definitive evidence of stability. Third, the endpoint was short-term functional visual outcome assessed at 4 weeks after the third injection, and minor variability in retrospective follow-up timing cannot be excluded; longer follow-up is needed to determine whether the model predicts long-term stability and recurrence. Fourth, OCT biomarkers were quantified manually on a single horizontal transfoveal B-scan, which may introduce subjective bias and may not fully capture spatial retinal heterogeneity. Finally, baseline BCVA was the dominant predictor. Although the full model outperformed the BCVA-only benchmark, the incremental value of the additional features remains modest and should be interpreted cautiously. Larger multicenter prospective studies with longer follow-up and more automated OCT quantification are needed to confirm generalizability and clinical utility.

## 5 Conclusion

In conclusion, this study developed and validated an interpretable XGBoost-based ML model integrating five key baseline features of BCVA, age, RVO subtype, DRIL, and SRF. The model showed encouraging performance for predicting poor functional visual outcomes in patients with RVO-ME after the anti-VEGF loading phase and maintained high sensitivity in the independent temporal test cohort. Furthermore, SHAP analysis suggested a non-linear relationship between baseline BCVA and prognosis, with a breakpoint at approximately 1.14 logMAR, and highlighted the independent predictive value of quantitative DRIL and SRF in determining functional visual recovery. Overall, this study provides a proof-of-concept for interpretable prediction in RVO-ME and may help identify patients at higher risk of poor short-term functional outcome. Larger multicenter studies are still needed to confirm its generalizability and clinical utility.

## Data Availability

The datasets presented in this article are not readily available because the raw data are restricted due to the conditions of the ethical approval, which only allows for the use of de-identified data for the purposes of this specific research. Requests to access the datasets should be directed to taoliming@ahmu.edu.cn.

## References

[1] Romano F Lamanna F Gabrielle PH Teo K Battaglia PM Iacono P . Update on retinal vein occlusion. Asia-Pac J Ophthalmol. (2023) 12:196–210. doi: 10.1097/APO.0000000000000598 36912792

[2] Vilela MA. Use of anti-VEGF drugs in retinal vein occlusions. Curr Drug Targets. (2020) 21:1181–93. doi: 10.2174/1389450121666200428101343, 32342813

[3] World Health Organization. World Report on vision. Geneva: World Health Organization (2019).

[4] Tsang KK Hui VWK Pang CMK Tang Z Yang D Nguyen TX . SD-OCT-based biomarkers in predicting treatment outcomes of macular oedema secondary to retinal vein occlusion treated with anti-VEGF therapy. Acta Ophthalmol. (2026) 104:e152–64. doi: 10.1111/aos.17574, 40757884 PMC12888950

[5] Ciulla TA Kapik B Hu A Harris A Ip MS Blodi B. Anatomic biomarkers of macular edema associated with retinal vein occlusion. Ophthalmol Retina. (2022) 6:1206–20. doi: 10.1016/j.oret.2022.06.016, 35781069 PMC9927025

[6] Weng SF Reps J Kai J Garibaldi JM Qureshi N. Can machine-learning improve cardiovascular risk prediction using routine clinical data? PLoS One. (2017) 12:e174944. doi: 10.1371/journal.pone.0174944, 28376093 PMC5380334

[7] Goldstein BA Navar AM Carter RE. Moving beyond regression techniques in cardiovascular risk prediction: applying machine learning to address analytic challenges. Eur Heart J. (2017) 38:ehw302–ehw1814. doi: 10.1093/eurheartj/ehw302, 27436868 PMC5837244

[8] Mehta N Modi Y Silva FQ Du W Moini H Singh RP. Machine learning to predict outcomes and dosing frequency with Aflibercept for macular edema following central retinal vein occlusion. Ophthalmic Surg Lasers Imag Retin. (2026) 57:212–8. doi: 10.3928/23258160-20251201-02, 41705990

[9] Schlosser T Beuth F Meyer T Kumar AS Stolze G Furashova O . Visual acuity prediction on real-life patient data using a machine learning based multistage system. Sci Rep. (2024) 14:5532. doi: 10.1038/s41598-024-54482-2, 38448469 PMC10917755

[10] Liang C Liu L Zhao T Ouyang W Yu G Lyu J . Predicting visual acuity after retinal vein occlusion anti-VEGF treatment: development and validation of an interpretable machine learning model. J Med Syst. (2025) 49:57. doi: 10.1007/s10916-025-02190-3, 40299116

[11] Ali S Akhlaq F Imran AS Kastrati Z Daudpota SM Moosa M. The enlightening role of explainable artificial intelligence in medical & healthcare domains: a systematic literature review. Comput Biol Med. (2023) 166:107555. doi: 10.1016/j.compbiomed.2023.107555, 37806061

[12] Schulze-Bonsel K Feltgen N Burau H Hansen L Bach M. Visual acuities "hand motion" and "counting fingers" can be quantified with the freiburg visual acuity test. Invest Ophthalmol Vis Sci. (2006) 47:1236–40. doi: 10.1167/iovs.05-0981, 16505064

[13] Chinese Vitreo-Retina Society of Chinese Medical Association, Fundus Disease Group of Chinese Ophthalmologist Association, Ophthalmology Group of China Alliance for Rare Diseases/Beijing Society of Rare Disease Clinical Care and Accessibility. Chinese expert consensus on the standardization of OCT and image interpretation and reporting in fundus diseases (2025): a Delphi approach. Zhonghua Yan Ke Za Zhi. (2025) 61:325–41. doi: 10.3760/cma.j.cn112142-20250108-00014, 40302593

[14] Peduzzi P Concato J Kemper E Holford TR Feinstein AR. A simulation study of the number of events per variable in logistic regression analysis. J Clin Epidemiol. (1996) 49:1373–9. doi: 10.1016/s0895-4356(96)00236-3, 8970487

[15] Grinsztajn L Oyallon E Varoquaux G. Why do tree-based models still outperform deep learning on typical tabular data? Adv Neural Inf Proces Syst. (2022) 35:507–20.

[16] Taunk K. De S. Verma S. Swetapadma A. A brief review of nearest neighbor algorithm for learning and classification. In 2019 International Conference on Intelligent Computing and Control Systems (ICCS), Madurai, India, (2019); pp. 1255–1260. doi: 10.1109/iccs45141.2019.9065747

[17] Suzuki M Nagai N Minami S Kurihara T Kamoshita M Sonobe H . Predicting recurrences of macular edema due to branch retinal vein occlusion during anti-vascular endothelial growth factor therapy. Graefes Arch Clin Exp Ophthalmol. (2020) 258:49–56. doi: 10.1007/s00417-019-04495-9, 31732812

[18] Sen P Gurudas S Ramu J Patrao N Chandra S Rasheed R . Predictors of visual acuity outcomes after anti-vascular endothelial growth factor treatment for macular edema secondary to central retinal vein occlusion. Ophthalmol. Retina. (2021) 5:1115–24. doi: 10.1016/j.oret.2021.02.008, 33610836 PMC8565966

[19] Chen T. Guestrin C. Xgboost: a scalable tree boosting system. In Proceedings of the 22nd acm Sigkdd International Conference on Knowledge discovery and data mining, San Francisco, CA, USA, (2016); pp. 785–794. doi: 10.1145/2939672.2939785

[20] Lundberg SM Erion G Chen H Degrave A Prutkin JM Nair B . From local explanations to global understanding with explainable AI for trees. Nat Mach Intell. (2020) 2:56–67. doi: 10.1038/s42256-019-0138-9, 32607472 PMC7326367

[21] Pappelis K Risi-Koziona A Agapitou C Korakas E Thymis J Pavlidis G . Retinal microvascular changes in association with endothelial Glycocalyx damage and arterial stiffness in patients with retinal vein occlusion: a cross-sectional study. Biomedicine. (2024) 12:2564. doi: 10.3390/biomedicines12112564, 39595130 PMC11592074

[22] Bonilha VL. Age and disease-related structural changes in the retinal pigment epithelium. Clin Ophthalmol. (2008) 2:413–24. doi: 10.2147/opth.s2151 19668732 PMC2693982

[23] Scott IU Campochiaro PA Newman NJ Biousse V. Retinal vascular occlusions. Lancet. (2020) 396:1927–40. doi: 10.1016/S0140-6736(20)31559-2, 33308475 PMC9546635

[24] Berry D Thomas AS Fekrat S Grewal DS. Association of Disorganization of retinal inner layers with ischemic index and visual acuity in central retinal vein occlusion. Ophthalmol. Retina. (2018) 2:1125–32. doi: 10.1016/j.oret.2018.04.019, 30511035 PMC6266856

[25] Vujosevic S Alovisi C Piccoli G Brambilla M Torti E Marenzi E . Severity of disorganization of retinal layers and visual function impairment in diabetic retinopathy. Ophthalmol Retina. (2024) 8:880–8. doi: 10.1016/j.oret.2024.04.005, 38604502

[26] Munk MR Ceklic L Stillenmunkes R Chaudhary V Waheed N Chhablani J . Integrated assessment of OCT, multimodal imaging, and cytokine markers for predicting treatment responses in retinal vein occlusion associated macular edema: a comparative review of anti-VEGF and steroid therapies. Diagnostics. (2024) 14:1983. doi: 10.3390/diagnostics14171983, 39272767 PMC11394301

[27] Babiuch AS Han M Conti FF Wai K Silva FQ Singh RP. Association of Disorganization of retinal inner layers with visual acuity response to anti-vascular endothelial growth factor therapy for macular edema secondary to retinal vein occlusion. Jama Ophthalmol. (2019) 137:38–46. doi: 10.1001/jamaophthalmol.2018.4484, 30286219 PMC6440246

[28] Daruich A Matet A Moulin A Kowalczuk L Nicolas M Sellam A . Mechanisms of macular edema: beyond the surface. Prog Retin Eye Res. (2018) 63:20–68. doi: 10.1016/j.preteyeres.2017.10.006, 29126927

[29] Murakami Y Notomi S Hisatomi T Nakazawa T Ishibashi T Miller JW . Photoreceptor cell death and rescue in retinal detachment and degenerations. Prog Retin Eye Res. (2013) 37:114–40. doi: 10.1016/j.preteyeres.2013.08.001, 23994436 PMC3871865

