# Supplementary material for: Interpretable machine learning to predict functional visual outcomes after the anti-VEGF loading phase for macular edema secondary to retinal vein occlusion: model development and temporal internal validation
Source: Front Med (Lausanne). 2026 May 18;13:1837014. doi: 10.3389/fmed.2026.1837014 (PMC13222776; doi:10.3389/fmed.2026.1837014)
Supplement: Supplementary file 1 [file Supplementary_file_1.docx]

Supplementary Table S1. **Definitions and standardized measurement methods of each OCT biomarker.**

| Biomarker | **Definitions** | **Standardized measurement methods** |
| --- | --- | --- |
| Vitreoretinal Abnormalities | The presence of pathological structural changes or abnormal traction at the vitreomacular interface on OCT images. | Evaluated qualitatively from OCT foveal scans. The interface is classified as abnormal ("Yes") if any of the following are observed: visible adhesion, incomplete vitreous detachment, complete posterior vitreous detachment, vitreomacular traction, or epiretinal membrane. Otherwise, it is classified as normal ("No"). |
| Central macular thickness (CMT) | The distance from the internal limiting membrane to the inner surface of the retinal pigment epithelium at the foveal level | CMT was measured using the built-in software of the OCT device. |
| The length of disorganization of the retinal inner layers (DRIL) | The state in which the boundary between any two of the ganglion cell-inner plexiform layer complex, inner nuclear layer, and outer plexiform layer cannot be identified within the horizontal OCT scan range | The length of DRIL was assessed across the full 6-mm width of the horizontal transfoveal B-scan at the foveal level using the built-in manual caliper tool. When DRIL was discontinuous, the lengths of all affected segments were summed. |
| The number of hyperreflective dots (HRD) | Small hyperreflective foci characterized by clear boundaries and a diameter of 20–40 μm | The number of HRD was counted by analyzing the foveal scan layer using Image J software. |
| The cross-sectional area of intraretinal fluid (IRF) | Accumulation of fluid within retinal layers (typically accompanied by retinal thickening), identified as hyporeflective spaces on OCT scans | The maximum cross-sectional area of IRF at the foveal level was measured using the built-in software of the OCT device. |
| The cross-sectional area of subretinal fluid (SRF) | Accumulation of fluid located between the outer surface of the photoreceptors and the inner surface of the retinal pigment epithelium (RPE) | The maximum cross-sectional area of SRF at the foveal level was measured using the built-in software of the OCT device. |
| The length of ellipsoid zone (EZ) disruption | Discontinuity or loss of the continuous hyperreflective EZ band on OCT foveal scans. | The length of EZ disruption at the foveal level was measured directly using the built-in software of the OCT device; if the EZ was completely continuous, the length was recorded as 0 μm. |

Supplementary Table S2. Inter-grader agreement for OCT-based parameters.

| OCT biomarker | Data type | Reliability metric | Agreement value |
| --- | --- | --- | --- |
| Vitreoretinal abnormalities | Categorical | Cohen’s kappa | 0.963 |
| Central macular thickness (CMT) | Continuous | ICC | 0.905 |
| DRIL length | Continuous | ICC | 0.702 |
| HRD count | Continuous | ICC | 0.571 |
| IRF cross-sectional area | Continuous | ICC | 0.713 |
| SRF cross-sectional area | Continuous | ICC | 0.804 |
| EZ disruption length | Continuous | ICC | 0.761 |

Note: Inter-grader agreement was assessed using Cohen’s kappa for categorical variables and intraclass correlation coefficients (ICC) for continuous variables.

Supplementary Table S3. Comparison of baseline characteristics between the included cohort and patients excluded due to incomplete loading phase.

|  | **level** | **Included Cohort** | **Excluded (Incomplete loading)** | **P value** |
| --- | --- | --- | --- | --- |
| n |  | 196 | 236 |  |
| Age, years (mean (SD)) |  | 59.56 (11.53) | 58.42 (11.86) | 0.313 |
| Sex (%) | Male | 96 (49.0) | 117 (49.6) | 0.902 |
|  | Female | 100 (51.0) | 119 (50.4) |  |
| Diagnosis (%) | CRVO | 76 (38.8) | 95 (40.3) | 0.754 |
|  | BRVO | 120 (61.2) | 141 (59.7) |  |

Supplementary Table S4. Comparison of baseline characteristics between the included cohort and patients excluded due to poor OCT quality

|  | **level** | **Included Cohort** | **Excluded (poor OCT quality)** | **P value** |
| --- | --- | --- | --- | --- |
| n |  | 196 | 181 |  |
| Age, years (mean (SD)) |  | 59.56 (11.53) | 61.42 (12.01) | 0.126 |
| Sex (%) | Male | 96 (49.0) | 93 (51.4) | 0.717 |
|  | Female | 100 (51.0) | 88 (48.6) |  |
| Diagnosis (%) | CRVO | 76 (38.8) | 109 (60.2) | < 0.001* |
|  | BRVO | 120 (61.2) | 72 (39.8) |  |
| BCVA (median [IQR]) |  | 0.92 [0.52, 1.30] | 1.30 [1.00, 1.70] | < 0.001* |

Supplementary Table S5. Hyperparameter search spaces and optimal configurations of the six machine learning models.

| Model | Hyperparameter | Search Grid / Range | Optimal Value |
| --- | --- | --- | --- |
| LR | - | - | - |
| SVM | C | 0.01, 0.1, 0.5, 1, 5, 10 | 1 |
| MLP | size | 1, 2, 3, 4, 5 | 4 |
|  | decay | 0.01, 0.1, 0.5, 1, 2 | 0.1 |
| KNN | k | 3 to 25 (step by 2) | 25 |
| RF | mtry | 2, 3, 4, 5 | 3 |
|  | ntree | Fixed at 500 | 500 |
|  | nodesize | Fixed at 5 | 5 |
| XGBoost | nrounds | 500, 1000 | 500 |
|  | max_depth | 2, 3 | 2 |
|  | eta | 0.01, 0.05 | 0.01 |
|  | gamma | 0.1, 1, 3 | 1 |
|  | min_child_weight | 3, 5 | 3 |
|  | colsample_bytree | 0.6, 0.8 | 0.8 |
|  | subsample | 0.7, 0.8 | 0.7 |

Note: LR, logistic regression; SVM, support vector machine; MLP, multilayer perceptron; KNN, k-nearest neighbors; RF, random forest; XGBoost, extreme gradient boosting.

Supplementary Table S6. Random-split sensitivity analysis and bootstrap-based stability estimation of model performance.

| Metric | Random-split sensitivity analysis, Mean ± SD | Bootstrap-based stability estimation, Result (95% CI) |
| --- | --- | --- |
| AUC | 0.899 ± 0.047 | 0.896 (0.767–0.992) |
| F1-score | 0.865 ± 0.045 | 0.897 (0.800–0.979) |
| Accuracy | 0.846 ± 0.048 | 0.876 (0.775–0.975) |
| Sensitivity | 0.877 ± 0.071 | 0.919 (0.792–1.000) |
| Specificity | 0.806 ± 0.068 | 0.808 (0.583–1.000) |

Note: Random-split sensitivity analysis was based on 10 repeated random 80/20 splits of the full cohort. Bootstrap-based stability estimation was performed using 1,000 resamples of the independent test set.


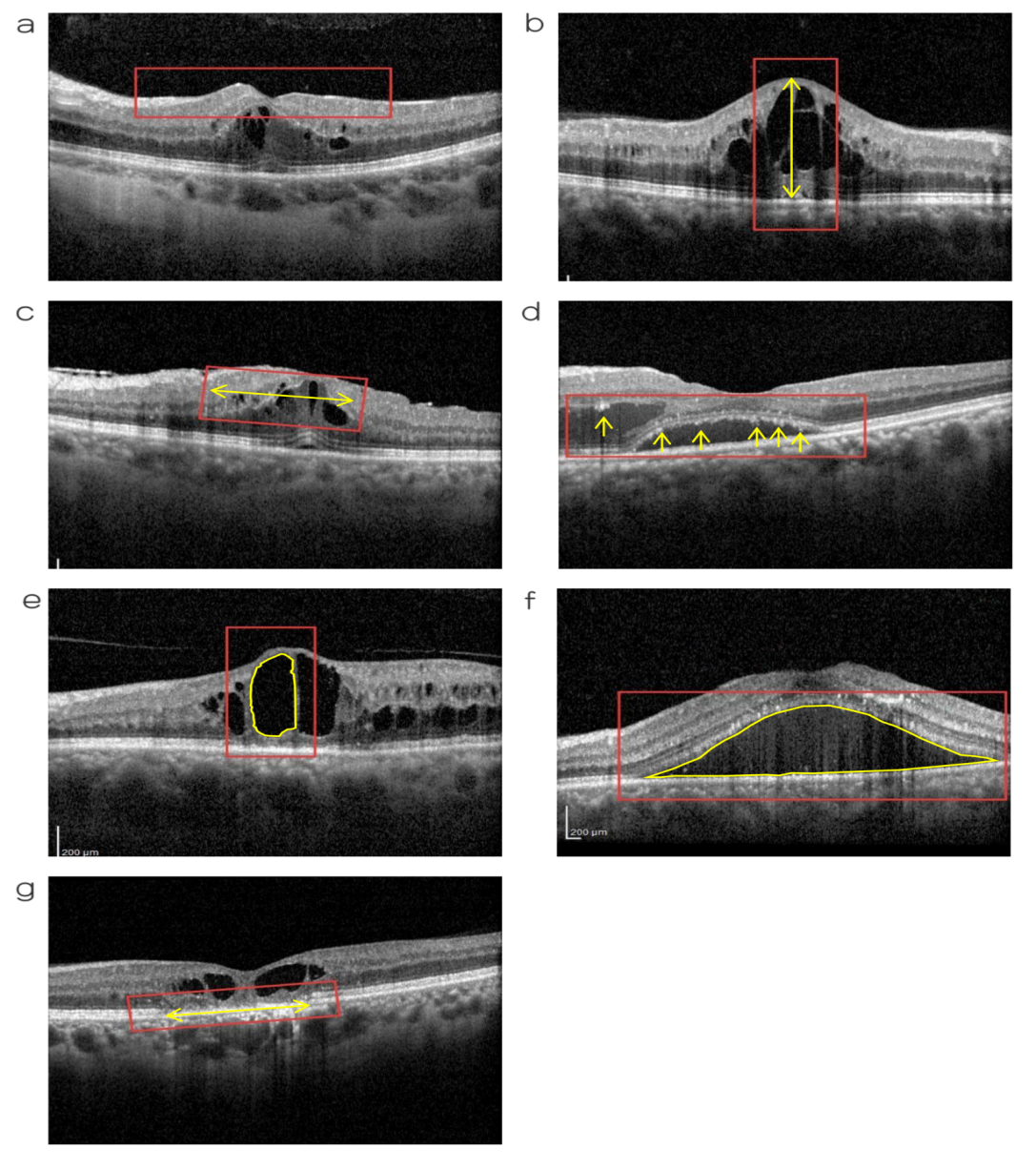


Supplementary Figure S1. Representative OCT images of biomarkers and standardized measurement protocols.

**(a)** Vitreoretinal Interface (Normal): A representative image of a normal vitreoretinal interface without visible adhesion, traction, or epiretinal membrane.

**(b)** Central Macular Thickness (CMT): Measurement of the distance between the internal limiting membrane (ILM) and the inner surface of the retinal pigment epithelium (RPE) at the foveal center.

**(c)** Disorganization of the Retinal Inner Layers (DRIL): Illustration of the horizontal extent where the boundaries between the inner retinal layers are indistinguishable.

**(d)** Hyperreflective Dots (HRD): Presence of small, discrete hyperreflective foci (yellow arrows) with diameters ranging from 20 to 40 μm.

**(e)** Intraretinal Fluid (IRF): Quantitative assessment of the maximum cross-sectional area of intraretinal fluid at the foveal level.

**(f)** Subretinal Fluid (SRF): Quantitative assessment of the maximum cross-sectional area of subretinal fluid at the foveal level.

**(g)** Ellipsoid Zone (EZ) Disruption: Measurement of the length of discontinuity in the hyperreflective EZ band at the foveal level.


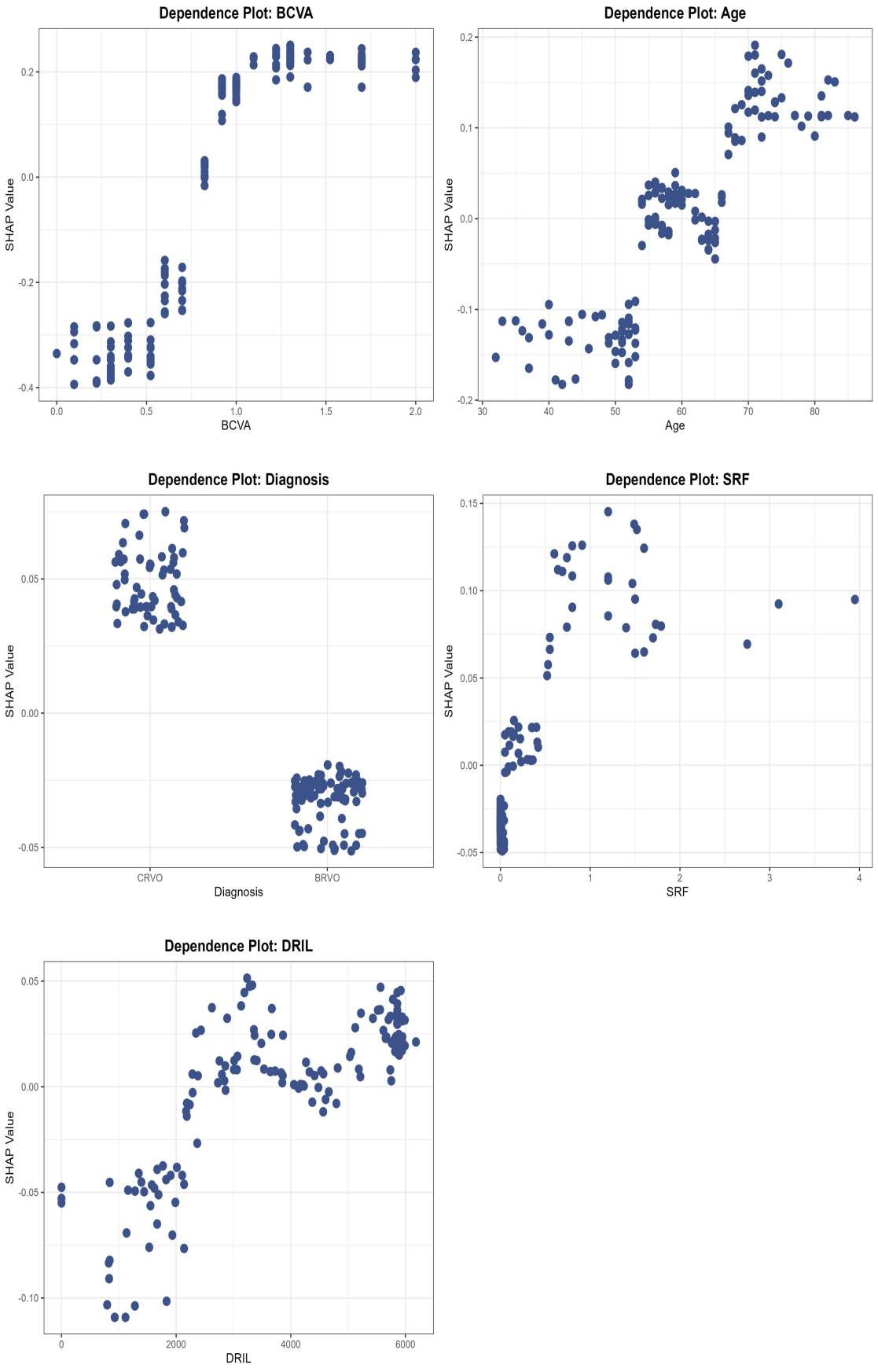


Supplementary Figure S2. SHAP dependence plots for the final predictors.
